# Advance zonal rectangular low energy adaptive clustering hierarchy algorithm for optimal routing in wireless sensors network

**DOI:** 10.1371/journal.pone.0321938

**Published:** 2025-05-30

**Authors:** Fazia Akhtar, Ijaz Ahmed, Ahmed F. Youssef, Idris H. Smaili, Mohamed Mostafa Ramdan Ahmed, Ali M. El-Rifaie

**Affiliations:** 1 Computer Science Department, Federal Urdu University of Arts, Science and Technology, Islamabad, Pakistan; 2 Interdisciplinary Research Center for Sustainable Energy Systems, King Fahd University of Petroleum and Minerals, Dhahran, Saudi Arabia; 3 College of Engineering and Technology, American University of the Middle East, Egaila, Kuwait; 4 Department of Electrical and Electronic Engineering, College of Engineering and Computer Science, Jazan University, Jazan, Saudi Arabia; 5 Department of Electrical Technology, Faculty of Technology and Education, Helwan University, Cairo, Egypt; Thamar University: Dhamar University, YEMEN

## Abstract

In wireless sensor networks (WSNs), power consumption is a recurring issue. Compared to other modern routing approaches that aim to reduce power consumption, cluster-based forwarding algorithms have been shown to be more energy efficient. Static clustering optimization is the main emphasis of this study on energy-efficient advanced zonal rectangular low energy adaptive clustering hierarchy (EE-AZR-LEACH) optimum routing, which takes a modern technique. To extend the lifespan of the cluster units and the system, we suggest using the multi-hopping approach. The proposed protocol significantly improves the network life time and energy efficiency of WSNs by optimizing static clustering and incorporating multi-hopping techniques. It can outperform existing protocols in power consumption, data transfer and stability, makes it a robust solution for large-scale and energy constrained environment. To help the Cluster Heads (CHs) with data transmission, EE-AZR-LEACH chose a Collaborator(CL) close to the central cluster. To increase the effectiveness of communication between the CHs located in the rectangular region and a central base station, these units took on the role of cluster leaders. The resilience, data transmission rate, power consumption, network endurance, and number of CHs of the system were clearly improved as a consequence. Our suggested routing system performs more effectively than AZR-LEACH, LEACH, MH-LEACH, and SEP in substantial areas. Furthermore, the proposed approach exhibits better convergence within 600 rounds when compared to AZR-LEACH, LEACH, MH-LEACH, and SEP. The findings indicate that after 1500 simulation cycles, the stability intervals for LEACH, MH-LEACH, SEP, and AZR-LEACH are 2.7%, 7.2%, 4.14%, and 5.34%, respectively. The simulation is run using MATLAB. The EE-AZR-LCH optimum routing, on the other hand, has a 6.8% survival rate. The MH-LEACH optimum routing has smaller total network tenure even if it provides a higher stability period than the EE-AZR-LEACH.

## 1 Introduction

A wireless sensor network (WSNs) is an assemblage of sensors strategically positioned in various areas to gather information about environmental variables and transmit it to a central hub. Protocols that enhance the longevity of sensor nodes (SNs) are favoured in adhoc networks [1]. Cluster-based setup is a method that improves a network’s self-organization and optimizes a WSNs by efficiently using power. This technique is particularly useful for monitoring activities where data from several streams come together at a specific data sink [2]. Three key challenges arise in the design and development of a WSNs clustering approach. 1. The selection of cluster heads (CH); 2. Determining the placement of CH. 3. Imposing limitations on each cluster’s size.The probability that depends on the round determines the selection of CH in LEACH when nodes launch with the same amount of energy. The Hybrid Energy-Efficient Distributed algorithm, known as HEED, modifies the probabilistic CH selection by incorporating power LEACH and distributed power clustering. These are specifically designed for heterogeneous energy scenarios, where nodes begin with varying energy levels. However, these options do not offer guarantees regarding the selection of high-energy CH or the uniformity of CH dispersion. The objective of this study is to provide a low energy approach using a clustering hierarchy method to solve the optimum routing energy problem for WSNs channels.

A WSNs typically consists of static, permanent nodes. It has persisted for some time [3, 4]. Researchers have conducted various types of research on the power usage of SNs, which are considered stationary nodes. Battery power is one of the main limitations of a mobile sensor network. As a result, the network’s durability and level of quality declined [5]. Understanding energy consumption is crucial for developing efficient protocols and algorithms that extend the network’s lifetime by optimizing energy usage, particularly for WSNs devices powered by batteries. This is crucial for applications requiring long-term monitoring or deployments in remote or harsh environments where battery replacement is impractical. As WSNs grows in size and complexity, managing the energy becomes increasingly difficult. Analyzing energy consumption patterns helps us design scalable solutions that can efficiently handle larger networks without compromising performance or reliability. For instance a free-weighting matrix strategy has been incorporated by utilizing the event-triggering communication protocols to reduce the data exchange loads within the network [6]. The authors of work [7, 8] applied event-triggering based communication protocols for switching and directed graph topologies, respectively. [9], utilized estimation framework considering the disturbances in WSNs. To enhance fog nodes’ capabilities, the authors of work [10] applied decision tree machine learning framework to reduce the power utilization of sensors, and to improve the throughput. [11, 12], presented the improved hybrid framework known as neural-based collaborative filtering for better settlement of services related problems in communication network. Energy constraints can directly improve overall network reliability, ensuring uninterrupted data collection and transmission. To handle these limitations, meta heuristic optimization shows promise in entertaining strict system-oriented constraints. Some applications of these approaches can be found in works [13–19]. Studying energy usage helps to optimize communication protocols, such as routing algorithms and data aggregation techniques, to minimize unnecessary transmissions, reduce overhead, and conserve energy. Optimizing energy usage can lower operational costs associated with maintenance and battery replacement, making WSNs more economically viable. In conclusion, energy consumption in WSNs is of paramount significance for prolong network lifetime, enhancing scalability, improving reliability, optimizing communication protocols, and cost-effectiveness.

The researchers proposed a cluster-based data transmission system as a means to conserve energy. LEACH [20], MH-LEACH [21], LEACH-ME [22], CBR-Mobile [23], and other cluster-based energy-efficient protocols are available. The energy-efficient CH of the mobile WSNs are identified using this clustering technique, which employs multiple criteria [24]. The proposed protocol, which is known as ECBR-MWSN, is an improved version of the MH-LEACH protocol. Its goal is to control the energy consumption of sensor network nodes in order to prolong their lifespan. The ECBR-MWSN method selects CH based on mobility, remaining power, and distance from the base station (BST) [25]. When the allotted time has passed, the BST selects the new CH using the suggested procedure. The study of different protocols shows that LEACH is a mechanism based on clumping that lowers the amount of energy used in sensing networks. However, its core parts are limited to a small area for collaboration, along with commands for setting up groups and process hubs like base station(BST) or CH. The random rotation of all the SNs around these hubs consumes energy. In order to broadcast data over large distances to the BST, the clustering data transmission method employs adjustable clusters and spinning CH to minimize transmit lengths for the majority of nodes. LEACH surpasses traditional clustering algorithms and distributes the machine’s power demand across all sensors. Additionally, LEACH has the capability to perform local computing within each cluster, thereby reducing the amount of data that requires forwarding to the BST. Although this is significantly less expensive than communication, the processing still consumes a lot of energy. After each round, LEACH reorganizes its nodes and again creates clusters. This technique is very time-consuming, as it involves an overhead on all CH for sending and receiving messages in each round, as well as the processing of energy calculations for selecting the next round of CH. In this instance, scalability entails data aggregation, load balancing, efficient resource use, and also routing systems can also incorporate clustering [26]. LEACH uses a randomized CH selection method, potentially leading to uneven cluster formation. Certain nodes may take on the role of CH several times in a row, which would rapidly drain their energy and lead to an early network collapse. In LEACH, cluster creation entails a large cost in terms of control messages sent back and forth between nodes, particularly in the early setup stage [20]. This overhead can use a significant amount of bandwidth and energy, which lowers the protocol’s overall efficiency. LEACH typically uses single-hop communication to connect SNs and CH. Although this method makes short-range communication easier and uses less energy, it may not perform well in cases with poorly dispersed nodes or large-scale networks. In circumstances involving dense deployment or large-scale networks, LEACH can encounter scaling problems. The number of nodes increases the overhead associated with cluster creation and maintenance, potentially leading to network bottlenecks and performance degradation. LEACH selects CH based on a predetermined probability threshold. This inability to adjust to changing node energy levels or dynamic network circumstances may result in subpar performance, particularly in highly dynamic situations. The hierarchical protocols which were suggested for energy-efficient routing in WSNs particularly LEACH and LEACH-based protocols are the main topic of this paper. This article compares LEACH theoretically to its descendent protocols using a variety of measures [27]. Through the use of simulation testing, the efficacy, LEACH is contrasted with three descendent protocols. In comparative [28] suggested method concentrates on dynamic cluster formation and CH selection using the Zebra Fish Optimization (ZFO) and Sea Horse Optimization (SHO) algorithms. A novel multi-level threading approach improves the ZFO algorithm, which uses a fitness function to dynamically choose the best CH. The SHO method then optimizes energy usage in the network with its novel adaptive parameter tuning approach. In other contemporary studies one approach integrated low energy adaptive clustering hierarchy algorithm with the analytic hierarchy process (AHP). This approach maintains a matrix within nodes incorporating the threshold values which presents the probability of a node to become a CH.[29] This method empowers the nodes to autonomously determine their probability of becoming the CH based on their energy status and distance to the Sink, eliminating the need for centralized control. In the comparative study of [30], leach-based routing algorithms are divided into five categories: algorithms that optimize CH selection, algorithms that optimize data transmission, algorithms that optimize both CH selection and data transmission, algorithms that employ fuzzy logic, and algorithms that maximize network energy by utilizing external energy sources. In work [31], authors suggested dual-phased meta heuristic-based frameworks for energy utilization in WSNs. The proposed approach provide a finer convergence on desired solution by handling communication constraints and more reliability compared to traditional optimization frameworks. By incorporating multi-hop communication, MH-LEACH may increase the network’s coverage and communication range, but it may also present scalability issues. Managing multi-hop routing pathways is more difficult as network size or node density rises, which raises overhead and increases the possibility of bottlenecks. MH-LEACH, like LEACH, can have uneven energy usage between nodes, especially when multi-hop communication is taking place.Relaying data from remote nodes to nodes adjacent to the sink or CH may lead to early energy depletion and a shorter network lifespan, as noted by Preethiya *et al*. [32]. Although the goal of multi-hop transmission in MH-LEACH is to increase energy efficiency by minimizing consumption of energy per hop and lowering transmission distance, real energy savings may differ based on interference, link quality, and routing path length. Poor routing choices or ineffective route management can counter multi-hop communication. In dense deployment situations or large-scale WSNs, SEP could encounter scaling issues. Network congestion, delay, and decreased throughput may result from the overhead involved with cluster creation, communication, and coordination growing with the number of nodes.

It is difficult for each CH to establish a direct connection from the base station when the deployment region of the sensors is rather large. Substantial transmission power is required to get the data through the CH to the base station whenever the base station is far from the CH. Since LEACH only assumes that every CH is one hop from the base station, it is not a suitable approach in this case. One approach that addresses this issue is MH-LEACH which is an enhancement of LEACH, reduces the energy consumption of the CH compared to large WSNs. During the setup process, cluster construction and choice of CH are done. The most feasible and energy efficient path is selected for that CH which is far from the base station. The method used for the selection of intermediate CH is the distance between the CH and the base station. The CH closer to the base station receives the data from the other CH which is far from base station. This helps to save energy of those CH which belong to the clusters with larger distance from the base station as higher transmission energy cost is required for communication with larger distances. Managing a large number of CH and maintaining synchronization among them can become increasingly complex and resource-intensive. SEP [33] introduces additional routing overhead in terms of control messages exchanged between sensor nodes, especially during the cluster formation phase and data transmission to CH. This overhead consumes energy and bandwidth, reducing the overall efficiency of the protocol and limiting its applicability to energy-constrained environments. In large-scale WSNs, the adaptive nature of AZR routing paths may pose scalability challenges. As the number of SNs increases or the network size increases, managing routing paths and energy monitoring becomes increasingly complex and resource-intensive, which adversely affects the network’s reliability and performance.

The goal of the energy efficient AZR-LEACH (EE-AZR-LEACH) optimal routing protocol is to optimize energy usage during cluster creation in each cycle by using a structured method with set and evenly sized clusters. The node with the highest energy level initiates a single instance of CH selection to enable the clusters.EE-AZR-LEACH is optimal Routing that uses a multi-hop paradigm and collaborative nodes, which helps to alleviate scalability issues that are present in protocols like LEACH, especially when large-scale and highly populated networks are involved. This multi-hop strategy also addresses congestion problems. By prioritizing nodes with the most remaining energy, CHAR streamlines the CH selection process, in contrast to certain protocols that need threshold processing. Because of its simple operating paradigm, it can easily adjust to changing network circumstances. Moreover, by integrating advanced nodes (AN) and collaborators (CL), CHAR addresses energy imbalance issues that are common in protocols like MH-LEACH, SEP, and LEACH. All things considered, EE-AZR-LEACH optimal routing successfully tackles a number of issues, including network sustainability, energy optimization, excessive control messages, scalability, flexibility, and the complexity of CH selection thresholds.

There are some limitations in the AZR-LEACH which is deployed in an area of 250 x 250 m, but for a more extensive area, it runs down in terms of

- Stabilization time due to lower life span of alive nodes.
- High energy consumption of CH because of more significant deployment network area of SNs. item Number of deceased nodes in the early hours.
- Smaller throughput because of the small life span of alive nodes.

The highlighted issues are addressed and resolved in this article. The contributions of our proposed technique EE-AZR-LEACH optimal Routing as follows:

- Lengthen the stabilization time of AZR-LEACH by increasing the life span of alive nodes.
- Decrease the number of dead nodes by increasing the life span of cluster nodes.
- Increase the throughput by boosting the number of packets received in AZR-LEACH.
- Optimize the overall energy consumption of AZR-LEACH. Increase the number of rounds for data transfer by any CH.
- Removed the overhead of message forwarding from the CH.
- Increased the life of CH due to less energy consumption.
- It reduced the time consumption for selecting a new CH due to the longer stable state of the CH.
- Increased the life of the whole network and Improved the energy efficiency of the protocols as well as the entire network.

## 2 Proposed system model

**Fig 1:**
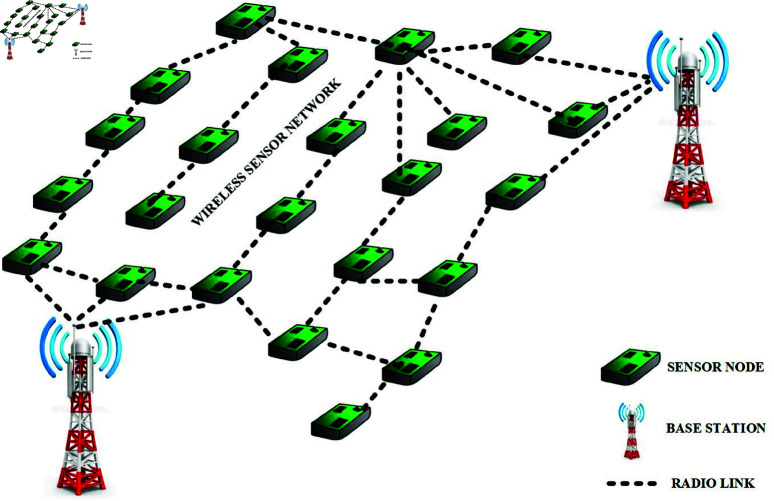
The fundamental layout of WSNs.

Fig 1 depicts the fundamental architecture of WSNs employed in this procedure. We assume that the SNs initially have homogeneous antenna gain, are planted erratically and evenly, and are not movable. It’s also believed that the sensor field’s dimensions are known as the base station’s locations. The BST may collect statistics from the CH, aggregate them, and then transfer them to the desired destinations. The LEACH protocol employs dynamic clustering. The EE-AZR-LEACH methodology simultaneously employs static clustering. The AZR-LEACH optimal routing technique is relatively simple and energy-efficient. It’s a turn-based procedure, similar to LEACH, and each round includes two phases: the initialization phase and the layout phase. Unlike LEACH, this protocol incorporates a rectangle-shaped cluster and a combined group of rectangular clusters that form zones.

### 2.1 Initialization phase

**Fig 2:**
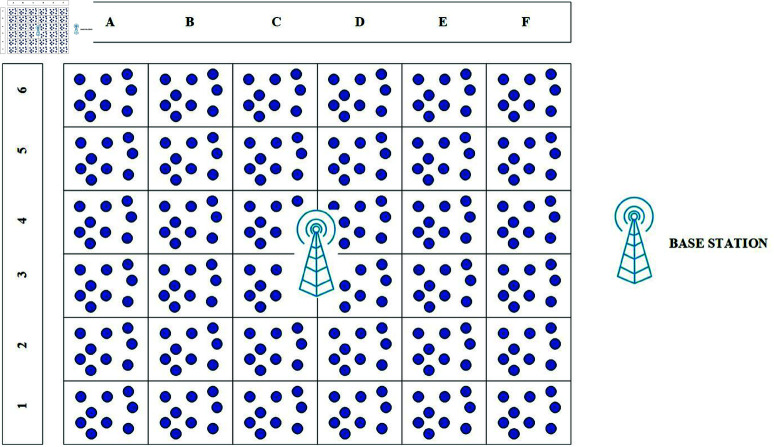
Rectangle shaped clusters arrangement.

This work organizes the entire network into fixed-sized rectangular-shaped clusters. These rectangular clusters are heterogeneous means the number of nodes in each bunch may vary. Typically, we position the BST near the center of the deployment area. The dimensions of the deployment region are required for the construction of rectangular clusters, as shown in Fig 2. We expect to identify the size of the deployment area and the location of the BST. The sensor area is divided from the centre outwards, and every rectangle-type cluster covers a similar amount of space. If the entire region is 1000 x 1000 m and the BST is at the midpoint, in this case, there could be two possibilities: either the cluster is so big because of which the distance between the nodes becomes bigger or the number of clusters will increase.

### 2.2 Formation of zones

**Fig 3:**
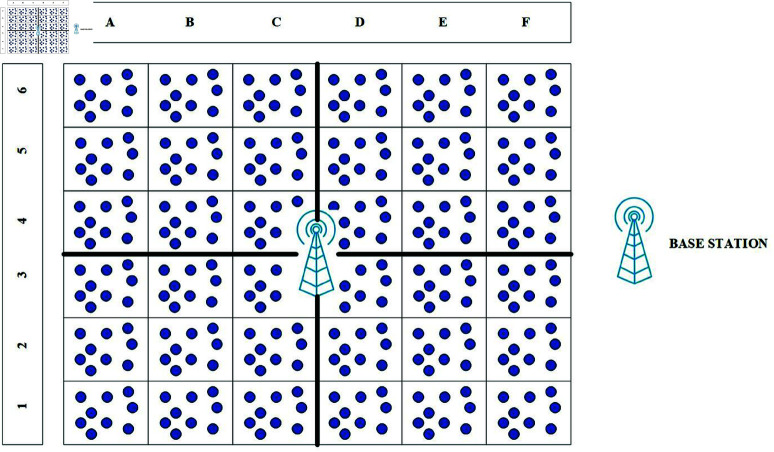
Formation of zones.

The adjacent cluster will be joined to make a zone. The purpose of zones is to organize and manage the nodes in a better way. In Fig 3. it is visible that clusters A1, A2, and A3, together with clusters B1, B2, and B3, along with clusters C1, C2, and C3, make Zone1. Zone2 which consists of the clusters A4, A5, and A6 with B4, B5, and B6 along with C4, C5, and C6. Zone3 have the clusters D1, D2, and D3 with clusters E1, E2, and E3 and F1, F2, and F3. In the same way, Zone4 is formed with clusters D4, D5, and D6, along with E4, E5, and E6, and F4, F5, and F6. The BST is at the midpoint of the employment region of the WSNs

### 2.3 Selection of advanced clusters

**Fig 4:**
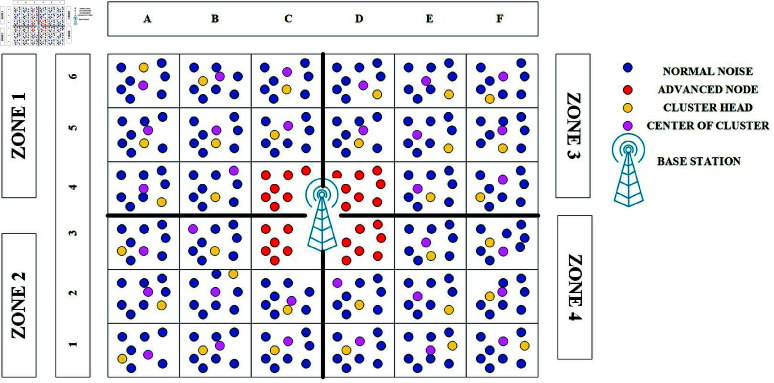
Selection of advanced nodes.

Fig 4 shows that Advance cluster (AC) have a direct connection to the BST, and AN are those nodes that are a part of such AC. The AN are illustrated in green. Advance cluster head (ACH) can receive data from nodes in their cluster and other clusters and transmit it to the BST. Advance cluster node (ACN) are used to facilitate multi-hop communication in a power proficient manner. Due to their proximity to the BST, the ACH utilize less transmission power than regular CH. In most cases, the BST is located in a reachable area so that AN can reach it more easily. Higher energy resources are also available in the AN. These consumed 1.5 times as much energy as the other nodes throughout our experiments. The stability area is increased by employing increased power in the AN, similar to SEP[34]. In essence, a CN nodes can only transmit data to its CH, and that head can only send it to the ACH of the latter’s specific zone.

### 2.4 Communication layout

**Fig 5:**
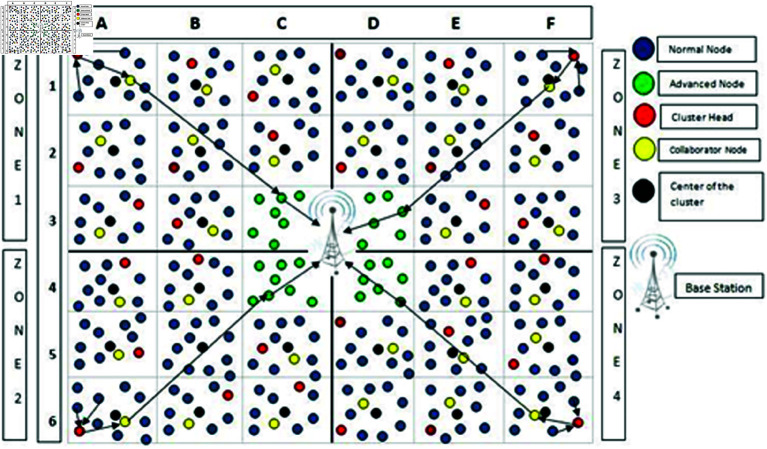
Cluster and communication layout.

In the setup phase for each cluster, the center is determined, and the node closest to it is chosen as the cluster CL, as shown in Fig 5. The CL broadcasts an NC packet containing the ID and lingering energy of the CL. When a node receives the packet, it compares its energy with the CL; if its energy surpasses the CL’s energy, it becomes the CH. The CH broadcasts a message to all the CN. For any cluster, if the energy of the CL goes below the threshold value (30% of the initial energy), then it broadcasts a MSG containing the ID and energy of the CL. When node X receives this packet from any node, it responds by sending a new packet to the CL. From the received packets, the node next to the CL with the utmost energy is selected as the new CL. At the same time, the node with the maximum energy and distance from the center becomes CH in the next round.

**Fig 6:**
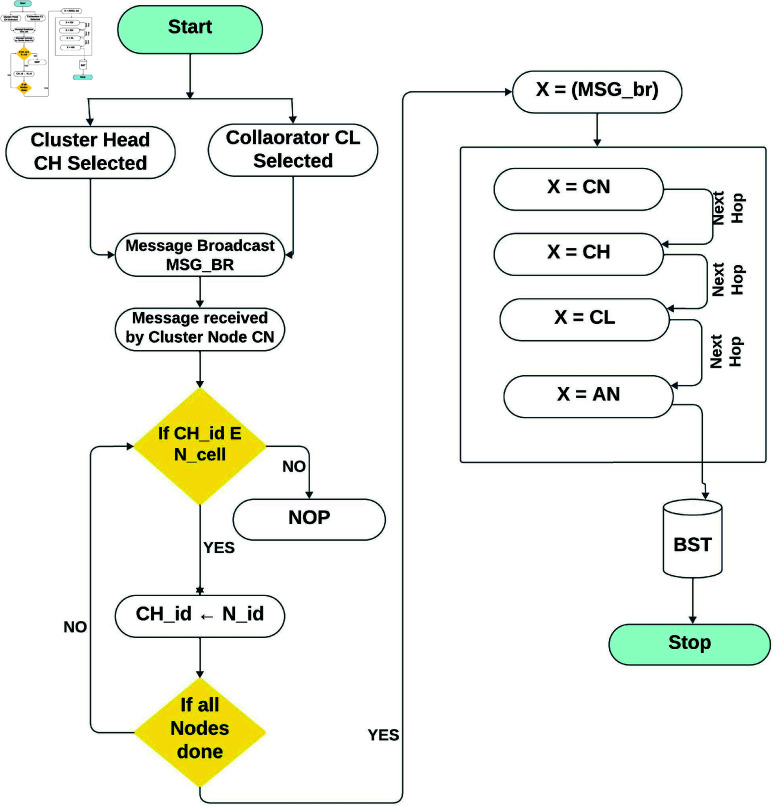
Work flow process of the proposed EE-AZR-LEACH protocol.

The new CH and the CL disseminate their status within their intra-cluster region via the CSMA/CA protocol. The network’s segmentation into defined rectangular clusters eliminates the need for CH to announce their standing to the entire system. Non-cluster head nodes that acquire an on-air message from their respective CH send a reply note back to the CH. The CH allocates TDMA intervals to every node in the bunch and publishes the TDMA agenda among all units in the cluster as shown in Fig 6.

### 2.5 Set point layout

After choosing CH and assigning TDMA slots, the set-point stage begins. The transmission between the CH and its associated nodes begins at their allocated time per TDMA procedure. Only a fixed time frame allows the CN to connect with its related CH. During unallocated time slots, all CN are in a sleep state. The set-point stage of the EE-AZR-LEACH optimal routing is a connection to the BST. The CH of each cluster receive statistics from the concerned CN, combine, and then compact all facts acknowledged by its cluster zone. The CH then forwards this data to the relevant cluster’s CL. The CL is responsible for forwarding the compressed facts to the ACH. The ACH then sends that data to the BST. This protocol is based on the following presumptions: each node can compute its power; each node is aware of its position; and all nodes can talk to the BST. The detailed workflow of the EE-AZR-LEACH protocol that visually represents the key steps of the protocol, from the initialization phase through to the data transmission is shown in Fig 6. Which illustrates the EE-AZR-LEACH protocol’s complete process. The figure consists of two phases which are as follows:

Phase 1: The selection of CH, describe the complete process of CH selection. the working of this layout stage is described in Table 1.

**Table 1:** Algorithm for layout stage.

Algorithm 1: Algorithm for Layout Stage
*CL*: Collaborator
*C*: center of the cluster
*CL*: Collaborator Node
*CN*: cluster Node
*N*_*Cell*: Node Cell
*Ch*_node: Child Node
1. Begin
2. (Loop) Repeat "*j*" for every cluster
3. Cent ← center of each cluster grid
4. *CL* ← *min*_*dist* (*C*, *N*, *E*_*max*_)
5. *MSG*_*BR*{*MSG*_*PAC*,*CL*_*id*,*E*_*CL*_}
6. After receiving the broadcast *msg* from the collaborator, any node compares the energy
7. if ($E_{x}\geq E_{CL}$)
a. *X* ←*CH*;
b. *MSG*_*BR*{*MSG*_*RES*,*CH*_*id*,*E*_*CH*_}
8. While any node *N* receives the message
9. If (*N* ←*N*_*cell* )
a. *MSG*_*BR*{*MSG*_*C*,*N*_*id*,*CH*_*id*}
10. While receiving *MSG*_*C* by the node having *CH*_*id*
11. If (*CH*_id←N_*cell* )
12. *Ch*_*node* ←*N*_*id*
13. End loop
14. End

Phase 2: The Layout Phase outlines the complete hierarchy of the communication and data flow. The working of this set point layout phase is described in the Table 2.

**Table 2:** Data propagation algorithm stages.

Algorithm 2: Data Propagation Algorithm in set point stage
*BST*: Base Station
*AN*: Advanced Node
*CL*: Collaborator Node
*CH*: Cluster Head
*CN*: Cluster Node
1. Begin
2. *br*_*msg*{ *BST*_*MSG*, *BST*_*id* , *BST*_*Loc*}
3. *X*(*br*_*msg*)
4. If (*X* is *AN*)
a. Next *hop AN* ← *BST*
b. Send it to *BST*
c. Goto step 3
5. Else if (*X* is *CL*)
a. Next *hop CL* ← *AN*
b. Send it to *AN*
c. Goto Step 4
6. Else if (*X* is *CH*)
a. Next *hop CH* ← *CL*
b. Send it to *CL*
c. Goto Step 5
7. Else if (*X* is *CN*)
a. Next *hop CN* ← *CH*
b. Send it to *CH*
c. Goto Step 6
8. If any data sensed by any node
a. Send data to its next *hop*
b. If (next *hop* is *BST*)
c. STOP the process
9. Else get to step 8
10. End

The overall time and space complexity of the algorithm are computed with the relation $N=O\left(\sum_{j=1}^{k}n_{j}\right)$, and complexity of the algorithm is as summing up the actions for every node *n*_*j*_, accounting all clusters yield the overall time complexity and *N* is the total numbers of node. The computed result for proposed approach is O(10404) for space complexity.

## 3 Experimental setup results and simulations

A 500 m x 500 m region randomly distributes the nodes. The BST is located in the middle of the deployment area, with coordinates of 500 m x 500 m. The simulation’s node population is composed of 1500 nodes (n = 1500). All the other contemporary protocols in literature such as LEACH, MH-LEACH, SEP, and AZR-LEACH used the deployment area as probably 100 m x 100 m but the proposed protocol gave a scalability of increased deployment area. The selected area reflects a small to medium size WSNs. It will finely work in remote areas where there is no infrastructure and the only possible communication is wireless. In these scenarios, this is a justified selection for the communication protocol. If the entire region is 1000 x 1000 m and the BST is in the midpoint. In this case, there could be two possibilities either the cluster is so big because of which the distance between the nodes becomes bigger or the number of clusters will be increased which also increases node density and the elevated energy consumption of the CH. Each simulation uses a distinct sample of randomly placed nodes, and the findings mentioned in the following sections are the mean values of 30 runs. The settings in Table 1. are used to simulate the LEACH, MH-LEACH, SEP, AZR-LEACH, and EE-AZR-LEACH best routing methods.

**Fig 7:**
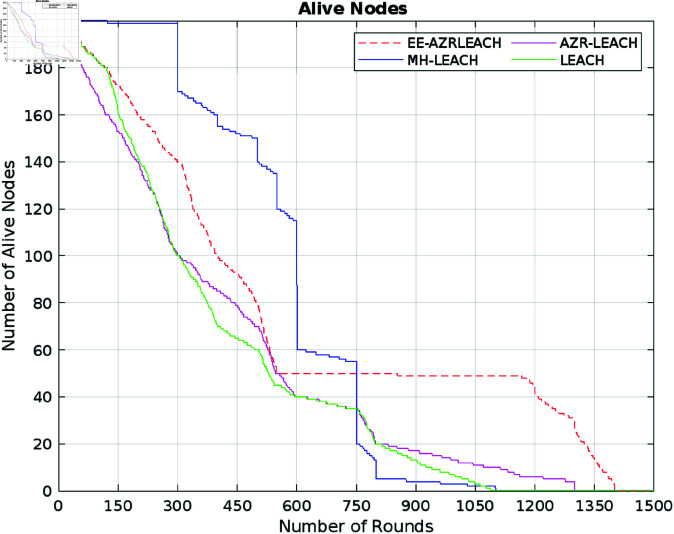
Number of alive nodes.

To acquire the simulated findings, MATLAB software is employed. As previously established, EE-AZR-LEACH optimal Routing functions in phases. 1500 cycles in total were utilized for our trials. To evaluate EE-AZR-LEACH optimal routing to AZR-LEACH, LEACH [5], MH-LEACH [24], and SEP [25], simulations of these four models are run. The findings are used to compute the recurrence of deceased and vibrant nodes every round, the statistic of CH vertices every round, the stability period, and the cumulative system performance. According to Fig 7, EE-AZR-LEACH opimal routing has a longer stabilization time than AZR-LEACH, LEACH [5], and SEP [25], but at a certain point, it has a shorter durability period than MH- LEACH. [24]. The primary unit of EE-AZR-LEACH optimal routing, expires after around 102 cycles, while the earliest nodes of LEACH, MH-LEACH, SEP, and AZR perish after roughly 39, 107, and 62 hoops, respectively. Considering all 1500 cycles, LEACH, MH-LEACH, SEP, and AZR-LEACH have stability intervals of 2.7%, 7.2%, 4.14%, and 5.34%, respectively, whereas EE-AZR-LEACH optimal routing has a survival time of 6.8%. Although the MH-LEACH offers a more extended period of stability than the EE-AZR-LEACH optimal routing, its total network lifespan is shorter. After around 1079, 1113, 1284, and 1401 rotations, accordingly, the last units of LEACH [24], MH-LEACH [24], SEP [25], and AZR-LEACH are deceased, while EE-AZR-LEACH’s last unit was terminated around 1450.

**Fig 8:**
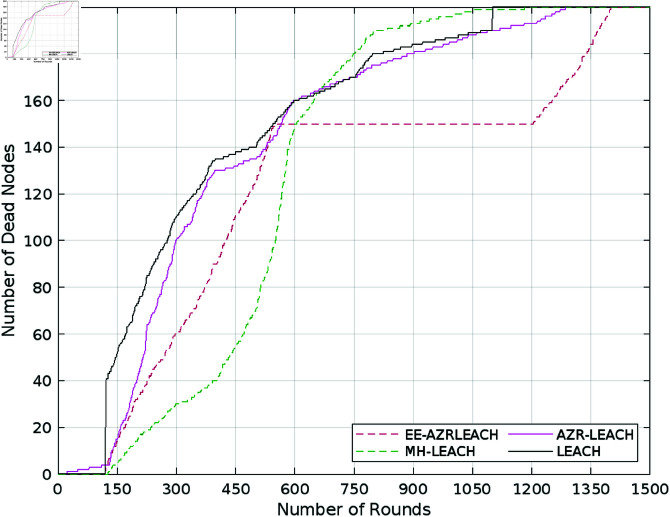
Number of dead nodes.

The stabilization time can be computed by analyzing the number of alive nodes, as shown in Fig 7. that it decrease with the lower count of these alive nodes. Due to the limited lifespan of these alive nodes, the throughput also decreases. Therefore, tracking the lifespan of these alive nodes can assist to extend the protocol’s stabilization time while simultaneously increasing throughput. On the other hand, monitoring dead nodes during the early phases of the network’s operation can have a direct impact on its stability. Calculating this number of dead nodes assists us in identifying the causes of early node failures, which may stem from the overhead in communication during the setup phase or the location of these nodes. Understanding these factors during crucial startup hours of the network’s lifecyle are critical for improving network stability and performance. So refereeing to Fig 8, the network lifespan of EE–AZR–LEACH optimal routing is 26%, AZR–LEACH is 24%, 21.56%, and 8.36% higher than those of LEACH, MH-LEACH, and SEP, accordingly.

**Fig 9:**
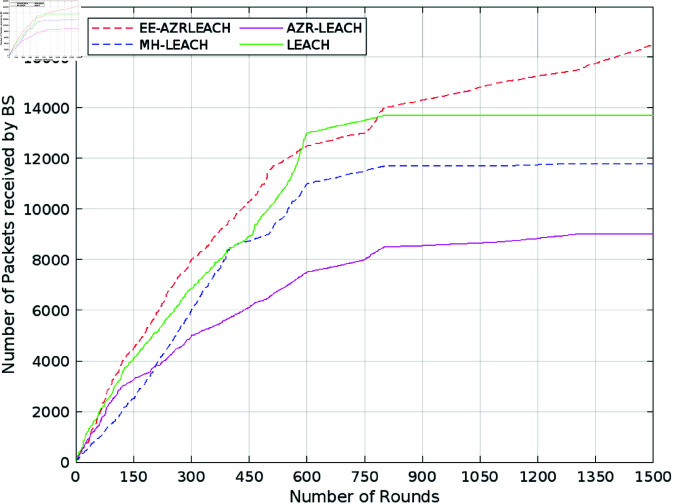
Number of packets received.

Fig 9 demonstrates that the productivity of EE-AZR-LEACH optimal routing in both stable and un-stable zones is much higher than that of LEACH, MH LEACH, SEP, and AZR-LEACH. In contrast to LEACH, MH LEACH, and SEP, EE-AZR-LEACH optimal routing ensures about 25%, AZR-LEACH is 23.49%, 31.61%, and 47.52% additional packets to the base station, accordingly. Due to its optimum frequency of cluster head picking and static grouping, EE-AZR-LEACH optimal routing has a higher throughput, especially in contrast to the other four techniques. Because of the time required for network setup, LEACH trades off latency and energy efficiency while SEP provided a trade-off between energy efficiency and network stability and MH-LEACH trades off between scalability and energy overhead. There is trade-off between energy efficiency and the communication latency. For energy optimization we include a multi-hop strategy which produces a significant improvement in the CH energy. In the result a more stable network is achieved which has prolonged alive node life intervals, more prolong time between the death of first and last sensor node and a better reception for the packets at the base station. As a result, it is demonstrated that EE-AZR-LEACH optimal routing has a greater throughput than LEACH, MH-LEACH, SEP, and AZR-LEACH. Several performance metrics are used to assess the performance of proposed method such as stability intervals, throughput, and node survival rates. The first one is stability period which is the amount of time interval that passes between the beginning of network operations and death of the first sensor node and usually represented by $T_{stable}=E_{int}/P_{avg}(N)$. The throughput which simply stated rate of data sent from member nodes to their respective cluster heads is computed using $TH_{CH\to \sin k}=D_{CH\to \sin k}/T_{CH\to \sin k}$, while the metrics of node survival is computed using $t_{s}=E_{int}/P$. Due to stationary clustering, EE-AZR-LEACH optimal routing and AZR-LEACH each have sufficient CH.

**Fig 10:**
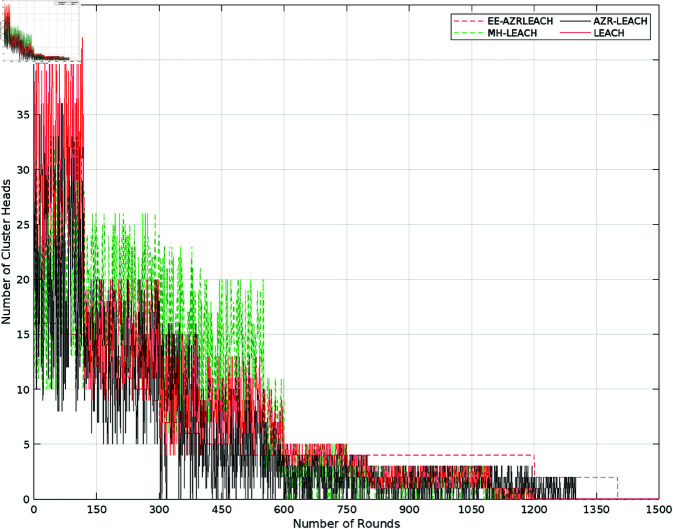
Number of CH and rounds.

In contrast to EE-AZR-LEACH optimal routing, which selects a set amount of CH per phase, LEACH, MH-LEACH, and SEP all use distributed methods to choose the number of CH, as shown in Fig 10. The choice of CH in LEACH, MH-LEACH, and SEP is unknown. When fewer CH are selected, the CH must transfer more data to member nodes, which causes the CH power to discharge more quickly. Once a node becomes CH, it must assume additional responsibilities. The network’s power utilization increases as the CH grow.

**Fig 11:**
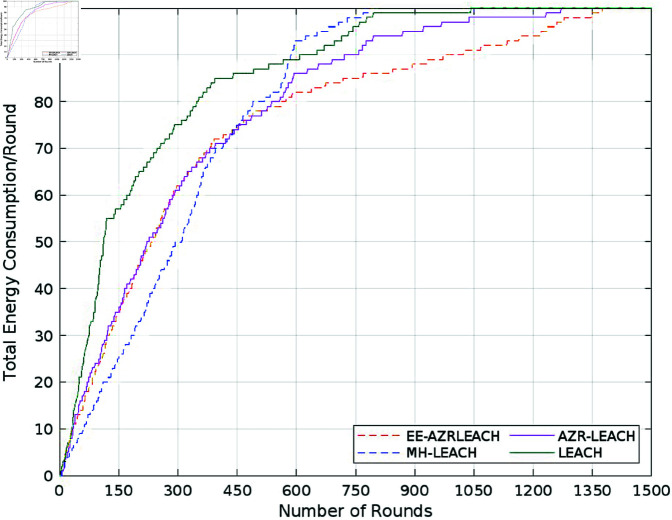
Total energy consumption.

The power utilization of LEACH, MH-LEACH, SEP, and AZR-LEACH with EE-AZR-LEACH optimal routing is compared in Fig 11. LEACH, MH-LEACH, SEP, and AZR-LEACH each require 100 joules of energy to achieve the threshold degree in 1078, 1112, 1282, and 1400 rounds, respectively. In 1450 rounds, the EE-AZR-LEACH optimal routing technique achieves a threshold scale of 100 joules. This demonstrates that our proposed approach consumes less energy than LEACH, MH-LEACH, and SEP by roughly 23%, 20.57%, and 8.42%, respectively.

A significant variation in energy use is also displayed in the performance table. According to the description, LEACH used upto 100 Joules of energy in 1078 rounds, MH-LEACH used it up in 1112 rounds, and SEP used it up in 1282 rounds. AZR-LCH terminated it after 1400 rounds, although it performed far better. By using 100 Joules up to 1450 rounds, EE-AZR-LEACH increases the number of feasible rounds. Its entire network life duration is increased to 1450 rounds due to this improved energy, which also improves the stability interval by 6.8%.

Additionally, as the performance Table 3. shows a visible difference in the energy consumption. It is clearly described that LEACH consumed 100 Joules energy in 1078 rounds; MH-LEACH depletes this energy in 1112 rounds while SEP utilized it completely in 1282 rounds. Though AZR-LEACH worked much better but it also ended it in 1400 rounds. EE-AZR-LEACH enhances the number of workable rounds and utilized 100 Joules up to 1450 rounds. Because of this optimized energy the not only the stability interval is enhanced by 6.8% but also its overall network life span is extended up to 1450 rounds. This makes the proposed protocol much reliable.

**Table 3:** Comparisons with other approaches.

Name of Algorithms	Stable Region (Death of First Node in Round)	Stability Intervals (considering 1500 rounds in %)	Network Life Span in Rounds	Throughput of EE-AZR-LCH comparison in percentage	Energy Utilization 100 Joules in Rounds
LCH	40	2.7	1079	23.39% more than LCH	1078
M-LCH	106	7.2	1113	31.51% more than M-LCH	1112
SEP	61	4.14	1284	47.42% more then SEP	1282
AZR-LCH	81	5.34	1401	20% more than AZR-LCH	1400
EE-AZR-LCH	102	6.8	1450	-	1450

## Discussion

Table 3 thoroughly compares our proposed approach with state-of-the-art protocols such as AZR-LEACH, LEACH, MH-LEACH, and SEP, taking into account crucial factors like throughput, energy consumption, and the number of dead nodes. The addition of Figs 7 to 11, which clearly illustrate the efficacy of our proposed EE-AZR-LEACH technique across a variety of metrics, has considerably enhanced this study’s contribution. The results unambiguously show a wide range in energy usage. 100 Joules of energy was utilized by LEACH in 1078 cycles. MH-LEACH used this energy in 1112 rounds, but SEP utilized it all in 1282 rounds. Even though it did much better, AZR-LEACH ended it after 1400 rounds. EE-AZR-LEACH employs 100 Joules up to 1450 rounds and increases the number of viable cycles to maximize network performance. Furthermore, a discernible variation in energy usage is displayed in the performance table. It is stated quite plainly that LEACH used up 100 Joules of energy in 1078 rounds, MH-LEACH used it up in 1112 rounds, and SEP used it up in 1282 rounds. AZR-LEACH terminated it after 1400 rounds, although it performed far better. By using 100 Joules up to 1450 rounds, EE-AZR-LEACH increases the number of feasible rounds.

## 4 Conclusion and future implications

We proposed an optimum forwarding strategy for WSNs. The main objective was to improve the CH choosing process. Our suggested method makes use of continuous clustering. For each cluster, we select the CH based on the remaining node power in EE-AZR-LEACH’s optimum routing. In the best EE-AZR-LEACH routing, the first node dies after 102 rounds, which is more than 62 rounds longer than LEACH, 61 rounds longer than SEP, and 21 rounds longer than AZR-LEACH, but less than four rounds shorter than MH-LEACH. With stability intervals of 4.1%, 0.4%, 2.66%, and 1.46%, respectively, EE-AZR-LEACH outperforms LEACH, MH-LEACH, and SEP. Optimal EE-AZR-LCH routing has a life duration that is 49 rounds longer than AZR-LEACH, 371 rounds longer than LEACH, 337 rounds longer than MH-LEACH, and 166 rounds longer than SEP. EE-AZR-LEACH has an optimal routing throughput that is 20% greater than AZR-LEACH, 23.39% higher than LEACH, 31.51% higher than MH-LEACH, and 47.42% higher than SEP. Energy consumption in 100 joules during EE-AZR-LEACH optimum routing rounds is 50 rounds higher than AZR-LEACH, 337 rounds higher than LEACH, 338 rounds higher than M-LEACH, and 168 rounds higher than SEP. It is determined that the EE-AZR-LEACH optimal routing in WSN, with its multi-hop communication, outperforms the current routing strategies (LEACH, MH-LEACH, SEP, and AZR-LEACH) in terms of network stability intervals, throughput, system lifespan, and energy consumption. When the suggested method is used, the simulated results show significantly improved performance in these parameters. EE-AZR-LEACH optimal routing is around 4% less efficient than MH-LEACH when dealing with large data traffic if the first node dies. By increasing the stability of EE-AZR-LEACH optimal routing, reaching such numbers extends the network’s sustainability.

In future, the work will be expended using distributed state estimation of WSNs considering wider area for deployment with system associated constraints.

## References

[1] Ismail A, Wang X, Hawbani A, Alsamhi S, Abdel Aziz S. Routing protocols classification for underwater wireless sensor networks based on localization and mobility. Wirel Netw. 2022;28(2):797–826.

[2] Balzano W, Stranieri S. A self-organization technique in wireless sensor networks to address node crashes problem and guarantee network connectivity. In: Web, Artificial Intelligence and Network Applications: Proceedings of the Workshops of the 33rd International Conference on Advanced Information Networking and Applications (WAINA-2019) 33. Springer; 2019. p. 841–50.

[3] Awwad S, Ng C, Noordin N, Rasid M. Cluster based routing protocol with adaptive scheduling for mobility and energy awareness in wireless sensor network. Proc Asia Pacific Adv Netw. 2010;20:57–65.

[4] Basit A, Tufail M, Rehan M, Ahmed I. A new event-triggered distributed state estimation approach for one-sided Lipschitz nonlinear discrete-time systems and its application to wireless sensor networks. ISA Trans. 2023;137:74–86. doi: 10.1016/j.isatra.2022.12.012 36588059

[5] Chandel A, Chouhan VS, Sharma S. A survey on routing protocols for wireless sensor networks. In: Advances in Information Communication Technology and Computing: Proceedings of AICTC 2019. Springer; 2020. p. 143–64.

[6] Ahmed I, Rehan M, Iqbal N, Basit A, Khalid M. Free-weighting matrix approach for event-triggered cooperative control of generic linear multi-agent systems: an application for UAVs. Arab J Sci Eng. 2024;49(5):6761–72.

[7] Basit A, Tufail M, Rehan M, Ahmed W, Radwan A, Ahmed I. Event-based secure filtering under two-channel stochastic attacks and switching topologies over wireless sensor networks. IEEE Trans Netw Sci Eng. 2024.

[8] Ali PR, Rehan M, Ahmed W, Basit A, Ahmed I. A novel output feedback consensus control approach for generic linear multi-agent systems under input saturation over a directed graph topology. ISA Trans. 2024. 10.1016/j.isatra.2024.02.029 38433069

[9] Basit A, Tufail M, Rehan M. An adaptive gain based approach for event-triggered state estimation with unknown parameters and sensor nonlinearities over wireless sensor networks. ISA Trans. 2022;129(Pt B):41–54. doi: 10.1016/j.isatra.2022.02.037 35341586

[10] Ullah R, Yahya M, Mostarda L, Alshammari A, Alutaibi AI, Sarwar N, et al. Intelligent decision making for energy efficient fog nodes selection and smart switching in the IOT: a machine learning approach PeerJ Comput Sci. 2024;10:e1833. doi: 10.7717/peerj-cs.1833 38660213 PMC11041942

[11] Ibrahim M, Bajwa I, Sarwar N, Waheed H, Hasan M, Hussain M. Improved hybrid deep collaborative filtering approach for true recommendations. Comput Mater Continua. 2023;74(3).

[12] Ibrahim M, Bajwa I, Sarwar N, Hajjej F, Sakr H. An intelligent hybrid neural collaborative filtering approach for true recommendations. IEEE Access. 2023.

[13] Ahmad H, Gulzar MM, Aziz S, Habib S, Ahmed I. AI-based anomaly identification techniques for vehicles communication protocol systems: Comprehensive investigation, research opportunities and challenges. Internet of Things. 2024;27:101245. doi: 10.1016/j.iot.2024.101245

[14] Ahmed I, Basit A, Ahmad M, AlMuhaini M, Khalid M. Electric mobility challenges and approaches for sustainable green power synergy in smart cities. Arab J Sci Eng. 2024;1–29.

[15] Ahmed I, Rehan M, Basit A, Malik SH, Ahmed W, Hong K-S. Adaptive salp swarm algorithm for sustainable economic and environmental dispatch under renewable energy sources. Renew Energy. 2024;223:119944. doi: 10.1016/j.renene.2024.119944

[16] Mustafa FE, Ahmed I, Basit A, Alqahtani M, Khalid M. An adaptive metaheuristic optimization approach for Tennessee Eastman process for an industrial fault tolerant control system. PLoS One. 2024;19(2):e0296471. doi: 10.1371/journal.pone.0296471 38381738 PMC10880964

[17] Ahmed I, Alvi U-E-H, Basit A, Khursheed T, Alvi A, Hong K-S, et al. A novel hybrid soft computing optimization framework for dynamic economic dispatch problem of complex non-convex contiguous constrained machines PLoS One. 2022;17(1):e0261709. doi: 10.1371/journal.pone.0261709 35081127 PMC8791528

[18] Ahmed I, Rehan M, Basit A, Tufail M, Hong K. Neuro-fuzzy and networks-based data driven model for multi-charging scenarios of plug-in electric vehicles. IEEE Access. 2023.

[19] Alvi U, Rehan M, Ahmed S, Ahmad R, Ahmed I. A novel incremental cost consensus approach for distributed economic dispatch over directed communication topologies in a smart grid. Soft Computing. 2022;26(14):6685–700.

[20] Alvarado G, Bosquez C, Palacios F, CoÂ´rdoba L. Low-energy adaptive clustering hierarchy protocol and optimal number of cluster head algorithm in a randomized wireless sensor network deployment. In: 2017 International Conference on Electrical, Electronics, Communication, Computer, and Optimization Techniques (ICEECCOT). 2017. p. 1–4.

[21] Panchal A, Singh L, Singh RK. RCH-LEACH: residual energy based cluster head selection in LEACH for wireless sensor networks. In: 2020 International Conference on Electrical and Electronics Engineering (ICE3). IEEE; 2020. p. 322–5.

[22] Das I, Das S. Energy efficient cluster analysis for heterogeneous wireless sensor networks. Wirel Personal Commun. 2021;121(1):337–52.

[23] Thandapani P, Arunachalam M, Sundarraj D. An energy-efficient clustering and multipath routing for mobile wireless sensor network using game theory. Int J Commun Syst. 2020;33(7):e4336.

[24] Rady A, Sabor N, Shokair M, El-Rabaie E. Efficient clustering based genetic algorithm in mobile wireless sensor networks. Menoufia J Electron Eng Res. 2020.

[25] Bhola J, Soni S, Cheema GK. Genetic algorithm based optimized leach protocol for energy efficient wireless sensor networks. J Ambient Intell Human Comput. 2019;11(3):1281–8. doi: 10.1007/s12652-019-01382-3

[26] Alyousuf RSM. Analysis and comparison on algorithmic functions of leach protocol in Wireless Sensor Networks WSN. In: 2020 Third International Conference on Smart Systems and Inventive Technology (ICSSIT). IEEE; 2020. p. 1349–55.

[27] Kandris D, Evangelakos EA, Rountos D, Tselikis G, Anastasiadis E. LEACH-based hierarchical energy efficient routing in wireless sensor networks. AEU – Int J Electron Commun. 2023;169:154758. doi: 10.1016/j.aeue.2023.154758

[28] Roberts M, Ramasamy P, Dahan F. An innovative approach for cluster head selection, energy optimization in wireless sensor networks using zebra fish and sea horse optimization techniques. J Indust Inf Integrat. 2024;100642.

[29] Gangal V, Cinemre I, Hacioglu G. A distributed leach-ahp routing for wireless sensor networks. IEEE Access. 2024.

[30] Hussain MHA, Mokhtar B, Rizk MR. A comparative survey on LEACH successors clustering algorithms for energy-efficient longevity WSNs. Egypt Inf J. 2024;26:100477.

[31] Roberts M, Thangavel J, Aldawsari H. An improved dual-phased meta-heuristic optimization-based framework for energy efficient cluster-based routing in wireless sensor networks. Alexandria Eng J. 2024;101:306–17.

[32] Preethiya T, Muthukumar A, Durairaj S. Double cluster head heterogeneous clustering for optimization in hybrid wireless sensor network. Wirel Pers Commun. 2019;110(4):1751–68. doi: 10.1007/s11277-019-06810-3

[33] Smaragdakis G, Matta I, Bestavros A. SEP: a stable election protocol for clustered heterogeneous wireless sensor networks. Boston University Computer Science Department; 2004.

[34] Garg S, Ghosh K. Energy allied routing in wireless sensor network. In: 2021 9th International Conference on Reliability, Infocom Technologies and Optimization (Trends and Future Directions) (ICRITO). IEEE; 2021. p. 1–5.

